# The Chemerin-CMKLR1 Axis is Functionally important for Central Regulation of Energy Homeostasis

**DOI:** 10.3389/fphys.2022.897105

**Published:** 2022-05-30

**Authors:** Haesung Yun, Rebecca Dumbell, Katie Hanna, Junior Bowen, Samantha L. McLean, Sriharsha Kantamneni, Klaus Pors, Qing-Feng Wu, Gisela Helfer

**Affiliations:** ^1^ School of Chemistry and Biosciences, Faculty of Life Sciences, University of Bradford, Bradford, United Kingdom; ^2^ School of Science & Technology, Nottingham Trent University, Nottingham, United Kingdom; ^3^ School of Pharmacy and Medical Sciences, Faculty of Life Sciences, University of Bradford, Bradford, United Kingdom; ^4^ Wolfson Centre for Applied Health Research, Bradford, United Kingdom; ^5^ Institute of Genetics and Developmental Biology, Chinese Academy of Sciences, Beijing, China

**Keywords:** chemerin, CMKLR1, bodyweight, energy homeostasis, hypothalamus, neuroinflammation, appetite

## Abstract

Chemerin is an adipokine involved in inflammation, adipogenesis, angiogenesis and energy metabolism, and has been hypothesized as a link between obesity and type II diabetes. In humans affected by obesity, chemerin gene expression in peripheral tissues and circulating levels are elevated. In mice, plasma levels of chemerin are upregulated by high-fat feeding and gain and loss of function studies show an association of chemerin with body weight, food intake and glucose homeostasis. Therefore, chemerin is an important blood-borne mediator that, amongst its other functions, controls appetite and body weight. Almost all studies of chemerin to date have focused on its release from adipose tissue and its effects on peripheral tissues with the central effects largely overlooked. To demonstrate a central role of chemerin, we manipulated chemerin signaling in the hypothalamus, a brain region associated with appetite regulation, using pharmacological and genetic manipulation approaches. Firstly, the selective chemerin receptor CMKLR1 antagonist α-NETA was administered i.c.v. to rats to test for an acute physiological effect. Secondly, we designed a short-hairpin-RNA (shRNA) lentivirus construct targeting expression of CMKLR1. This shRNA construct, or a control construct was injected bilaterally into the arcuate nucleus of male Sprague Dawley rats on high-fat diet (45%). After surgery, rats were maintained on high-fat diet for 2 weeks and then switched to chow diet for a further 2 weeks. We found a significant weight loss acutely and inhibition of weight gain chronically. This difference became apparent after diet switch in arcuate nucleus-CMKLR1 knockdown rats. This was not accompanied by a difference in blood glucose levels. Interestingly, appetite-regulating neuropeptides remained unaltered, however, we found a significant reduction of the inflammatory marker TNF-α suggesting reduced expression of CMKLR1 protects from high-fat diet induced neuroinflammation. In white and brown adipose tissue, mRNA expression of chemerin, its receptors and markers of adipogenesis, lipogenesis and brown adipocyte activation remained unchanged confirming that the effects are driven by the brain. Our behavioral analyses suggest that knockdown of CMKLR1 had an impact on object recognition. Our data demonstrate that CMKLR1 is functionally important for the central effects of chemerin on body weight regulation and neuroinflammation.

## Introduction

Homeostatic regulation of food intake and energy expenditure is a complex process, resulting from the interaction of hormones and neuromodulators in the hypothalamus of the central nervous system. The hypothalamus integrates peripheral signals, such as adipocyte-derived hormones (adipokines), to regulate appetite homeostasis. Although the mechanisms underlying the contribution of adipokines to appetite regulation are poorly understood, dysregulation plays a critical role in the development of obesity related complications.

Chemerin is an adipokine, encoded by the gene Rarres2, involved in inflammation, adipogenesis, angiogenesis and energy metabolism (Helfer and Wu 2018). Chemerin has been proposed as a link between obesity and the development of type-2 diabetes (Ernst and Sinal 2010; Gu et al., 2019). In mice, plasma levels of chemerin are upregulated by high-fat feeding (Ernst et al., 2010) and gain and loss of function studies show an association of chemerin with body weight, food intake and glucose homeostasis independent of diet (Ernst et al., 2012; Wargent et al., 2015). Chemerin is primarily produced in adipose tissue and circulating levels are correlated with visceral adipose tissue mass, waist circumference and body mass index in humans (Bozaoglu et al., 2007; Sell et al., 2009; Chakaroun et al., 2012). Visceral and subcutaneous expression of chemerin increases with obesity (Chakaroun et al., 2012) and local effects of chemerin on adipose tissue have been demonstrated in the proliferation of adipose tissue through hyperplasia (Jiang et al., 2018) and angiogenesis (Kaur et al., 2010) although conflicting results have been found in mouse mutant models (Helfer and Wu 2018).

Chemerin activates three known receptors: chemokine-like receptor 1 (CMKLR1), G protein coupled receptor 1 (GPR1), and chemokine (CC-motif) receptor-like 2 (CCRL2). These receptors are all involved in regulating metabolism and/or cell proliferation including glucose homeostasis, adipogenesis, energy balance, and inflammation (Helfer and Wu 2018). CMKLR1 is predominantly expressed in the liver, central nervous system, and white adipose tissue (WAT) where the chemerin-CMKLR1 signaling pathway is involved in energy homeostasis and inflammation response (Perumalsamy et al., 2017; Helfer and Wu 2018; Kennedy and Davenport 2018). GPR1 is expressed in similar tissues/cells to CMKLR1, and equally activates ERK1/2-MAPK kinases (De Henau et al., 2016). It is still unknown which proteins or kinases bind to CCRL2 but it has been suggested that its role is to increase local chemerin levels (De Henau et al., 2016). Interestingly, chemerin is highly expressed in obese and/or type-2 diabetes animal models (Sanchez-Rebordelo et al., 2018). Both CMKLR1 and GPR1 receptors play a critical role in insulin secretion from the β-cells of the pancreas indicating that chemerin is involved in insulin resistance and glucose uptake (Takahashi et al., 2011; De Henau et al., 2016). However, it is not clear whether energy/glucose homeostasis is controlled via the chemerin-CMKLR1 and/or the chemerin-GPR1 pathway and generally, it is still debated whether chemerin is involved in the control of glucose homeostasis per se (Léniz et al., 2022). Some studies have reported that CMKLR1-, GPR1-and chemerin-knockout mice show glucose intolerance (Takahashi et al., 2011; Ernst et al., 2012; Rourke et al., 2014; Wargent et al., 2015) whereas other studies show little to no effect on glucose homeostasis in mouse-mutant models (Rouger et al., 2013; Gruben et al., 2014).

Like other adipokines such as leptin, chemerin has proinflammatory effects that are likely to be important in its central actions. Several studies in humans have shown that serum chemerin levels are positively correlated with pro-inflammatory cytokines such as tumor necrosis factor alpha (TNF-α) and interleukin-6 (IL-6) (Léniz et al., 2022). In CMKLR1-knockout mice, Tnf-*α* and Il-6 mRNA levels are decreased in WAT (Ernst et al., 2012). In regards to the brain, it is well established that high-fat diet induces neuroinflammation in animal models of obesity, particularly in the hypothalamus, and leads to upregulation of inflammatory markers including nuclear factor-kappa B (NF-κB), TNF-α and IL-6 (Miller and Spencer 2014; Tan and Norhaizan 2019). Additionally, high-fat feeding decreases brain-derived neurotrophic factor (BDNF) which protects the hypothalamus from neuroinflammation (Ramalho et al., 2018). However, a link between hypothalamic inflammation and chemerin has not been established yet.

CMKLR1 has been identified as a functional receptor for the amyloid-β (Aβ42). Aβ42 accumulation reduces neuronal cell to cell communication in the brain and can lead to loss of function and neuronal death. Aβ42 activates CMKLR1, leading to glia cell migration and clearance of amyloid-β peptide suggesting the potential role of CMKLR1 in Aβ42 clearance and improvement in cognitive ability (Peng et al., 2015). In support, central administration of the nonapeptide chemerin-9, derived from the C-terminus of the chemerin precursor, enhances cognition and memory performance. Additionally, chemerin-9 protects against neuroinflammation caused by amyloid-β and memory impairment caused by Aβ42 (Lei et al., 2020). Furthermore, in a mouse model of Alzheimer’s CMKLR1 deficiency improves cognitive deficits (Zhang et al., 2020).

Given that high-fat feeding increases circulating levels of chemerin, induces inflammatory markers and results in a decline of cognitive performance (Helfer and Wu 2018; Tan and Norhaizan 2019; Léniz et al., 2022) and recent studies have implicated the chemerin-CMKLR1 axis in cognitive function (Lei et al., 2020; Zhang et al., 2020), in this study we aimed to test the link between the hypothalamic chemerin-CMKLR1 pathway, neuroinflammation and cognition after high-fat feeding. We used a combination of pharmacological and genetic manipulation approaches to block CMKLR1 signaling in the hypothalamus and showed that modulating chemerin function has a profound effect on body weight of high-fat fed rats acutely and chronically. Interestingly, hypothalamic chemerin-CMKLR1 signaling does not act directly through appetite regulating neuropeptides but alters the inflammatory marker TNF-α, suggesting that blocking CMKLR1 might protect from neuroinflammation. Markers of adipogenesis, lipogenesis and brown adipocyte activation in adipose tissue do not change, confirming that the effect is restricted to the brain.

## Materials and Methods

### Ethics Statement

All animal procedures were approved by the Animal Welfare Ethical Review Body at the University of Bradford and performed according to the Animals (Scientific Procedures) Act, 1986. Animal experiments were licensed by the United Kingdom Home Office (project license number: P0D6AA50D).

### Animal Experiments

**FIGURE 1:**
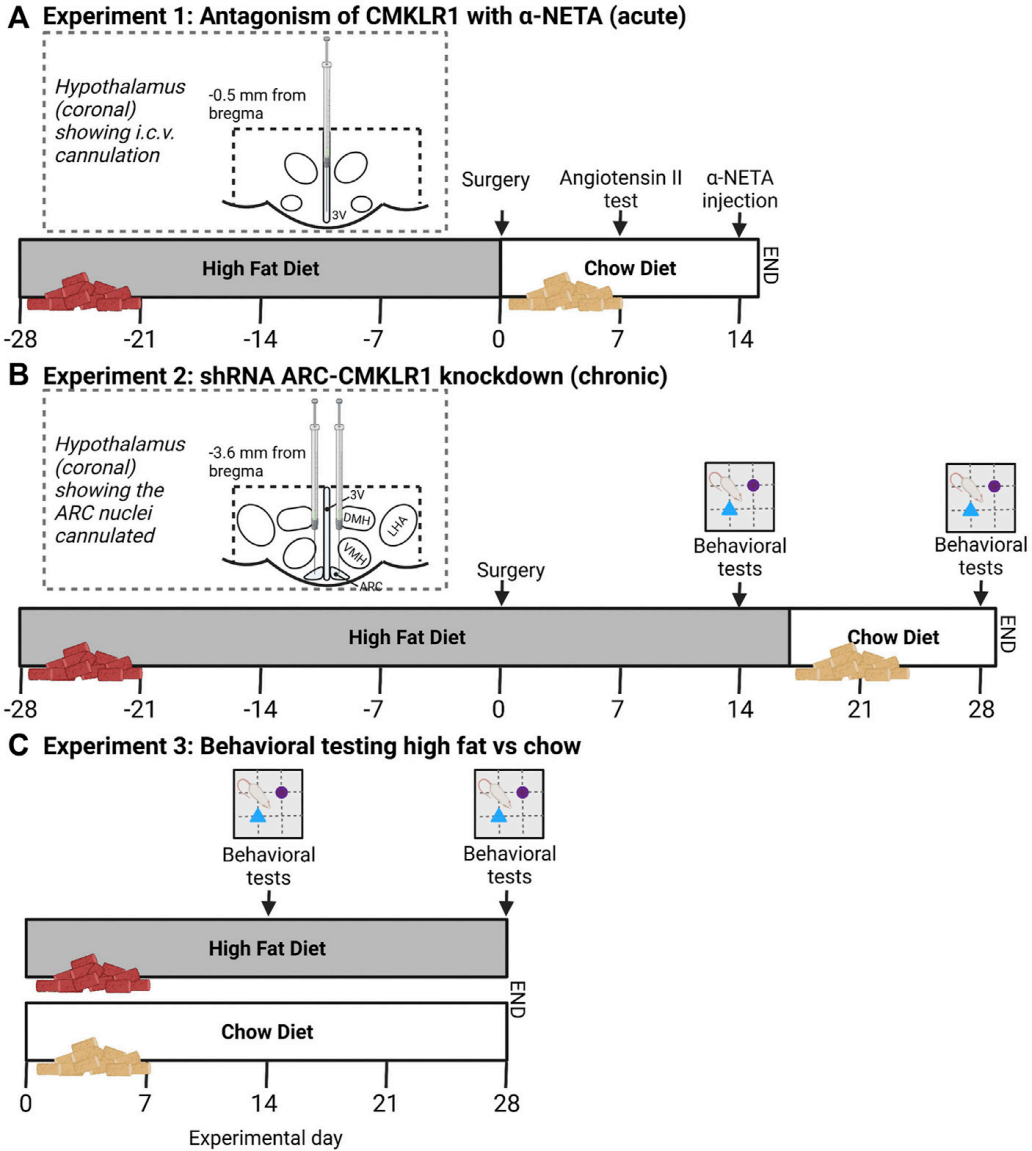
Schematic study design and timeline. **(A)** In experiment 1, Sprague Dawley rats received a high-fat diet for 28 days before i.c.v. cannulation. After surgery, rats were put on a chow diet. 7 days after surgery correct cannula placement was confirmed via Angiotensin II injections. 14 days after surgery, rats received a bolus injection of α-NETA, a selective CMKLR1 antagonist and body weight and food intake was measured 24 h after injection. **(B)** In experiment 2, lentivirus vectors expressing shRNA targeted against CMKLR1 (ARC-CMKLR1-KD) or scrambled virus was bilaterally injected into the arcuate nucleus (ARC) of Sprague Dawley rats on high-fat diet for 28 days. Diet was switched to chow diet after 17 days following surgery. Body weight was measured daily. Behavioral testing was carried out at experimental days 14 and 28. The inserts show the diagrammatic representation of the hypothalamus (coronal sections) showing the cannulated coordinates. The dotted lines bordering the hypothalamus indicate the hypothalamic area that was dissected for gene analysis. **(C)** In Experiment 3, Sprague Dawley rats were divided into two groups, one group was provided *ad libitum* with high-fat diet and the other group received nutrient matched chow diet. Rats were tested in the Novel object recognition (NOR) after 14 and 28 days of intervention to provide baseline behavioral data (created with Biorender.com).

Male Sprague Dawley rats were obtained from Envigo (Oxon, United Kingdom). Initially, rats were acclimatized for 7 days under 12 h light:12 h dark photoperiod in groups of four with *ad libitum* access to water and standard chow diet (2018 Teklad global 18% protein rodent diet, Envigo). After acclimatization, rats received a high-fat diet (45% fat by kcal, TD.06415, Envigo) or a nutrient-matched chow diet (10%fat by kcal, TD.06416, Envigo) depending on the experimental design (Figure 1). Rats were housed in standard rat cages (Type III rat caging, 2017 cm^2^ floor area, Arrowmight, Hereford, United Kingdom) with soft woodchip bedding (EC06 chips, Datesand, Manchester, United Kingdom) and a red plastic tunnel and shredded paper (Sizzle Nest, Datesand) for enrichment. All environmental conditions were kept constant with a temperature of 21 ± 10°C, humidity of 50 ± 5% and average light intensity of 150 lux. Body weights of individual rats and food intake of the cage was measured regularly at the beginning of the light phase as described in detail in (Mclean et al., 2021). Health checks were carried out twice daily and no welfare-related issues were observed. Animals were assigned random numbers before killing to allow all analyses to be conducted blind to the groupings.

Experiment 1 Antagonism of CMKLR1 with 2-(*α* naphtyl) ethyltrimethylammonium iodide (α-NETA)

Intracerebroventricular (i.c.v) cannulation and i.c.v. injections were carried out as previously described (Helfer et al., 2013). Briefly, after 4 weeks of high-fat feeding, rats (10–11 weeks of age, weight range 267.8–332.3 g) received 2.5 ml of 0.9% saline for circulatory support and 5.0% w/v Carprieve (Norbrook^®^ Labaratories, Newry, Northern Ireland). Rats were then anesthetized by inhalation (2.5–3% isoflurane) and were placed in a Kopf stereotaxic frame (David Kopf, New York, NY). EMLA Cream 5% (Lidocaine 25mg, Prolocaine 25 mg/g; Astrazeneca, Cambridge, United Kingdom) was applied to the periosteum, before a permanent 22-gauge stainless steel guide cannula (Plastics One Inc., Roanoke, VA, United States) was implanted into the third ventricle of the hypothalamus on the mid-sagittal line, 0.5 mm posterior to the bregma line and 6.5 mm below the outer surface of the scull. Cannulae were fixed with dental cement (AgnTho’s, Lindingoe, Sweden) to three stainless steel screws (AgnTho’s) inserted into the cranium. After post-operative recovery, rats were single housed. To allow injections under conscious conditions, rats were handled daily pre- and post-surgery and habituated to the injections process. Compounds were injected in a volume of 5 µl over 1 min using a 28-gauge stainless steel injection (Plastics One) projecting 1 mm below the tip of the cannula. Correct i.c.v cannula placement was confirmed by a positive dipsogenic response to angiotensin II (100 ng in 5 µl of saline; Sigma-Alrich, Poole, United Kingdom). One week after angiotensin II injection, rats were randomly divided into control group and α-NETA injection group and received either 5 µl of saline or 9.2 ng α-NETA (Sigma Aldrich) in 5 µl of saline over 1 min, respectively. I.c.v. injections were performed in the early light phase at 3–4 h after lights on (10:00–11:00; ZT3 - 4) with food intake and body weight measured shortly before and 1, 2, 4 and 24 h post injection. One rat was removed from data analysis as the cannula was blocked during the injection process, thus final animal numbers were: control group *n* = 8 and α-NETA injection group *n* = 6. Rats were killed 24 h after the i.c.v. injection at ZT3–4 by decapitation following isoflurane inhalation (Figure 1A). Guide cannulae were carefully removed and brains were immediately dissected, and the hypothalamus was frozen on dry ice and stored at −80^о^C.

Experiment 2 shRNA ARC-CMKLR1 knockdown

To block chemerin signaling in the ARC, a recombinant mammalian shRNA knockdown lentiviral vector was designed to inhibit the expression of rat CMKLR1 protein. CMKLR1 shRNA (sequence: 5′- GGA​AAG​CCA​TGT​GCA​AGA​TTA -3′) or control shRNA (sequence: 5′- CCT​AAG​GTT​AAG​TCG​CCC​TCG -3′) was inserted into the lentivirus vector pLV [shRNA]-EGFP:T2A:Puro-U6 under the control of the U6 promoter. A dual marker, EGFP fused to puromycin via T2A linker was inserted into the vector which is expressed under the control of the Human phosphoglycerate kinase 1 (hPGK1) promoter. The lentiviruses were named pLV-EGFP:T2A:Puro-U6>rCmklr1 [shRNA#4] and pLV-EGFP/Puro-U6>Scramble_shRNA. pLV-EGFP:T2A:Puro-U6>rCmklr1 [shRNA] and control vectors were constructed and packaged by VectorBuilder Inc. (Chicago, United States). The viral prep was concentrated (>10^9^ TU/mL) and ultra-purified (in HBSS) suitable for *in vivo* injection by VectorBuilder Inc. The efficiency to reduce expression of CMKLR1 was tested *in vitro* using rat primary cortical neurons cultured from E18 Sprague Dawley embryos before *in vivo* injections.

At 10–11 weeks of age (weight range 254.6–347.4 g), rats were randomly divided into groups and general surgical procedures were followed as described above. 3 μl of lentivirus vector expressing shRNA targeted against CMKLR1 (ARC-CMKLR1-KD, *n* = 8) or 3 μl of scrambled virus (control, *n* = 8) were bilaterally injected into the ARC using a micro syringe: 3.6 mm posterior to the bregma line, ±0.30 mm lateral to the mid-sagittal line, 9.8 mm below the outer surface of the skull. Stereotactic coordinates were calculated using the rat brain atlas of Paxinos and Watson (Paxinos and Watson 2005). After surgery, rats remained on high-fat diet for the first 17 days, but due to delivery problems of the high-fat diet, rats were then moved to nutrient-matched chow diet (10%fat by kcal, TD.06416, Envigo). Body weight and food intake were measured daily. Rats were killed 28 days after surgery, trunk blood was collected for serum and plasma and tissues (hypothalamus, white adipose tissue (WAT), brown adipose tissue (BAT)) were dissected and immediately frozen on dry ice and stored at −80^о^C. Glucose levels were measured in non-fasted rats from trunk blood after killing using a blood glucose monitoring system (OneTouch^®^ Select Plus, LifeScan UK&Ireland, United Kingdom) (Figure 1B).

Experiment 3 Behavioral testing high-fat vs. chow

18 male Sprague Dawley rats were obtained from Envigo (Oxon, United Kingdom) at 4–5 weeks old. After acclimatization rats (weight range 171.9–220.9 g) were randomly assigned to two groups of eight rats. One group was provided with *ad libitum* access to water and high-fat diet and the other group received chow diet. Rats were housed in groups of 4–5/cage to avoid effects of isolation on behavior. Rats were tested in the Novel object recognition (NOR) task after 14 and 28 days of diet intervention. After 29 days, rats were killed by terminal anesthesia using isoflurane followed by decapitation at ZT3 (Figure 1C).

### Gene Expression Analysis

Hypothalamic total RNA was isolated using the PureLink RNA mini kit (Invitrogen, United Kingdom) or PARIS™ kit (Thermo Fisher Scientific, United Kingdom) with on-column DNAse treatment. The hypothalamus was cut to approximately 60–80 mg per sample, transferred to cell disruption buffer and homogenized with 1.5 mm homogenization beads (Triple-Pure™, Molecular Biology Grade Zirconium Beads) using the BeadBug microtube homogenizer. After homogenization, the supernatant was transferred to Eppendorf tubes and RNA extraction was carried out according to the manufacturer’s protocols. The RNA was quantified using a Nanodrop™ spectrophotometer (Nanodrop Lite, Thermo Fisher Scientific, United Kingdom). 500 ng of RNA was used to synthesize the cDNA using iScriptTM cDNA synthesis kit (Bio-Rad, United Kingdom). qPCR was performed on an Eco™ Real-Time PCR system (Illumina Inc., CA, United States) to amplify gene expression levels using following parameters: 40 cycles of 2 min at 50°C, 5 min at 95°C, 10 s at 95°C, and 30 s at 60°C. mRNA levels were normalized to D-Box and fold changes were calculated using the 2^−ΔΔCt^ method. The sequences of each primer set were designed using NCBI gene sequences (Supplementary Table S1). Standard curves were performed for each primer set to confirm qPCR efficiency.

For the WAT and BAT gene expression analysis, RNA was extracted using Qiagen lipid tissue kit (Qiagen, Valencia, CA, United States), treated with DNase I (Qiagen) and cDNA synthesized according to kit instructions (Promega, Southampton, United Kingdom). qPCR was carried out on 56.25 ng cDNA on a QuantStudio™ 7 Flex Real-Time PCR System (Applied Biosystems) with PowerTrack™ SYBR Green Master Mix (Applied Biosystems). Relative expression was calculated by 2^-ΔΔCT method, using *β-Actin* (Actb) as the housekeeping gene and expressed relative to the scrambled control virus group.

### Behavioral Analysis

18 male rats were tested in the NOR task as previously described (Mclean et al., 2021). Rats were habituated to the test box for 20 min on 3 days. On the day of testing, rats were placed in the NOR chamber (52 cm wide × 40 cm high × 52 cm long) for 3 min. In the acquisition trial, rats were permitted to explore two identical objects for 3 min, they were then taken out of the NOR chamber and returned to their home cage for 1 min intertrial interval. During the intertrial interval, both objects were removed and the objects and the NOR chamber were cleaned with 70% EtOH. Rats were then exposed to a familiar object and a novel object for 3 min during the retention trial. The location of the novel object was randomly assigned using a Gellerman schedule. Acquisition and retention trials were recorded using a video recording system (Home Guard CCTV Home Security Kit). At the end of the experiments, the exploration time (sec) spent at each object in each trial was analyzed by an experimenter blind to the groups using two stopwatches. Rats that failed to explore both objects in one or both trials of the task were excluded from the analysis.

### Statistics

Group sizes were calculated based on gene expression analysis in surgical prepared rats from previous studies (Helfer et al., 2016). A sample size of seven to eight animals per group was found to provide the appropriate power to identify significant differences (*α* = 0.05). Body weight, food intake and energy intake data were analyzed by two-way repeated measures ANOVA (treatment group × time interaction). Area under the curve (AUC) of energy intake was calculated by setting the baseline as the mean of the first timepoint data for both groups and compared by two-tailed unpaired Student’s *t*-test. Gene expression was analyzed by two-tailed unpaired Student’s *t*-test or Mann-Whitney test as appropriate (MW: WAT Adipoq, Dio2, Cebpb, Cmklr1, Rarres2. BAT: Rarres2). The NOR data are expressed as mean exploration time ±SD. Data passed normality (Shapiro-Wilk) and student’s two-tailed paired *t*-test was performed to compare time spent exploring the familiar versus the novel object. Differences were considered statistically significant if *p* < 0.05. Data are presented as mean ± SD, n refers to the number of animals.

## Results

### Blocking CMKLR1 Acutely Reduces Body Weight and Food Intake

**FIGURE 2:**
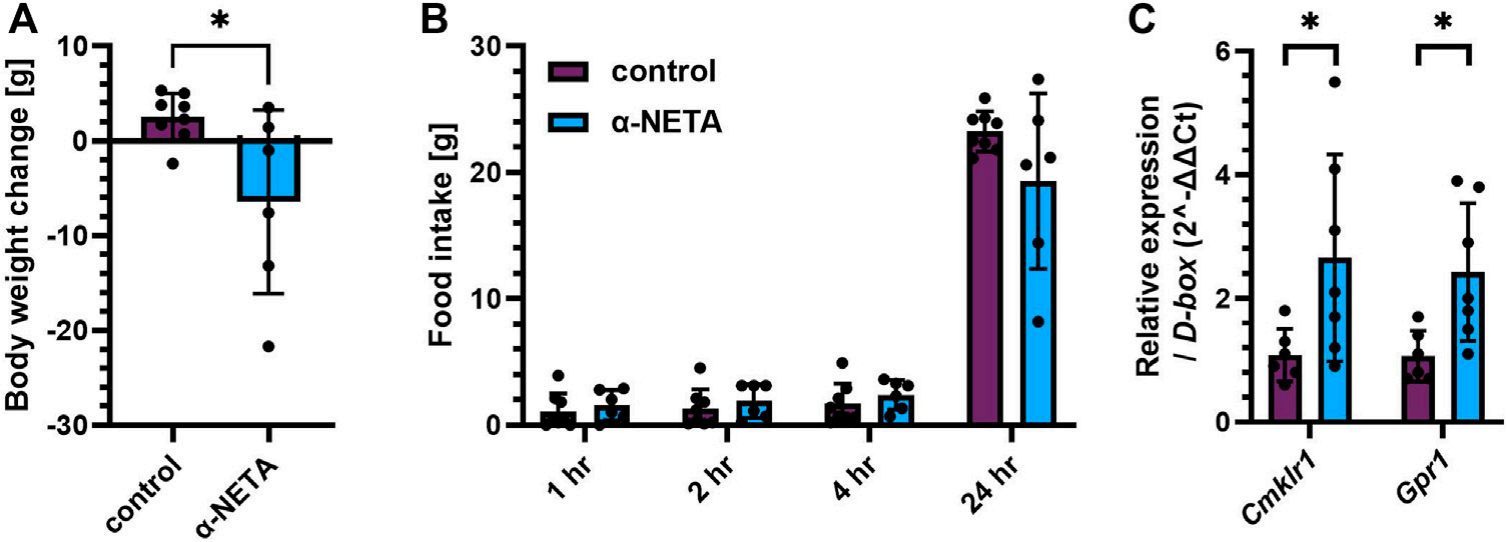
Blocking CMKLR1 with α-NETA reduced body weight and increased Cmklr1 and Gpr1 expression. High-fat diet (45%) fed Sprague Dawley rats received an i.c.v. injection of 5 µl saline (control, *n* = 8) or 9.2 ng in 5 µl α-NETA (*n* = 6) **(A)** Body weight change and **(B)** food intake was measured 1, 2, 4 and 24 h after injection. **(C)** Hypothalamic Cmklr1 and Gpr1 mRNA expression was measured by qPCR. Data are presented as mean ± SD. Data were analyzed by Student’s unpaired *t*-test. *p < 0.05.

To evaluate whether the small molecule CMKLR1 antagonist α-NETA (Graham et al., 2014) has the potential to regulate body weight and food intake *in vivo*, 5 µM α-NETA was administered i.c.v. into high-fat fed Sprague Dawley rats as a bolus injection. Acute injection of α-NETA caused a marked reduction in body weight (t_(12)_ = 2.526, *p* = 0.0266; Figure 2A). The reduction in body weight was accompanied by a decrease in food intake after 24hrs, however this was not significant (Figure 2B). Next, we tested whether α-NETA selectively targets Cmklr1 and Gpr1 in the hypothalamus. Interestingly, we found that both Cmklr1 and Gpr1 were significantly upregulated in α-NETA-injected rats compared to saline-injected rats (t_(11)_ = 2.230, *p* = 0.0476, t_(11)_ = 2.813, *p* = 0.0169, respectively; Figure 2C). Given these results, we decided to block CMKLR1 using a genetic manipulation approach.

### Weight Gain Is Inhibited in Arcuate Nucleus-CMKLR1-Knockdown Rats

**FIGURE 3:**
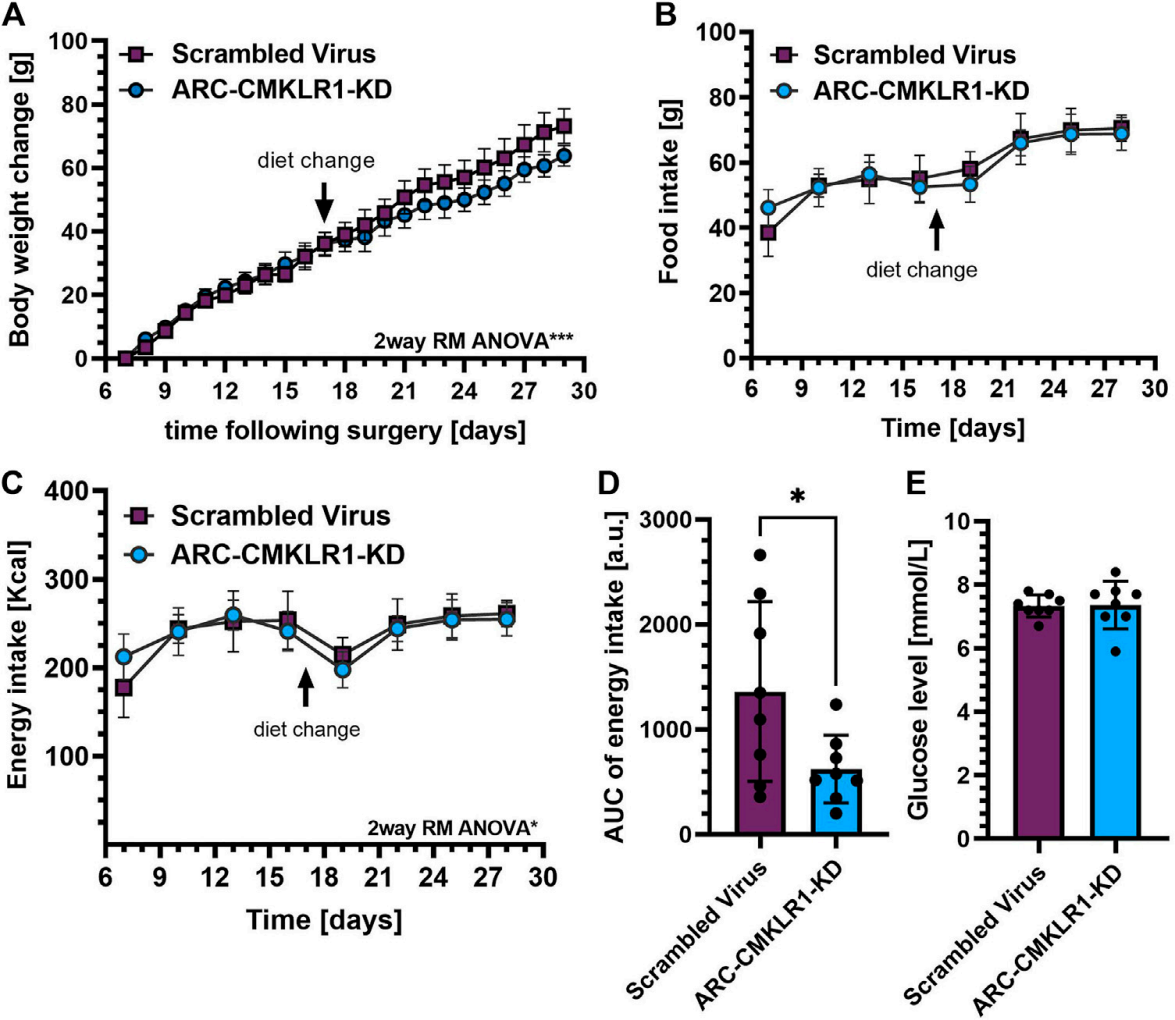
Effect CMKLR1 knock down in the arcuate nucleus on body weight, glucose levels, food and energy intake. Adult Sprague Dawley rats on high-fat diet received a bilateral injection of a shRNA construct targeted against CMKLR1 or a control construct (*n* = 8/group). After surgery, the rats were maintained on a high-fat diet for 17 days and then switched to chow diet. **(A)** Body weight change was calculated as the difference between body weights measured after post-operative recovery (day 7 after surgery) and 29 days after surgery showing that weight gain was inhibited in ARC-CMKLR1-KD rats (time x treatment, *p* = 0.001). **(B)** Food intake in 72 h showed no difference but **(C)** 2way RM ANOVA revealed a significant difference in energy intake (time x treatment, *p* = 0.0125) and **(D)** this was confirmed by analyzing the area under the energy intake curve. **(E)** No significant difference was found in unfasted glucose levels 29 days following surgery. Data are presented as mean ± SD.

Previously, we showed that i.c.v. administration of chemerin into rats altered food intake and body weight (Helfer et al., 2016). To demonstrate that this is achieved via the chemerin-CMKLR1 axis, we manipulated chemerin signaling in the arcuate nucleus of the hypothalamus. We designed a short-hairpin-RNA (shRNA) lentivirus construct targeting expression of CMKLR1 and tested for efficiency to reduce expression of this receptor RNA *in vitro* in rat primary cortical neurons. The shRNA construct, or a control construct was injected bilaterally into the arcuate nucleus of adult Sprague Dawley rats on high-fat diet. After surgery, these rats were maintained on high-fat diet for 2 weeks and then switched to chow diet for a further 2 weeks. Weight gain was inhibited in ARC-CMKLR1-KD rats (time x treatment, F_(22,308)_ = 2.300, *p* = 0.001) and this became apparent following the change in diet (Figure 3A). While there was no difference detected in food intake (Figure 3B), a small but statistically significant difference in energy intake between the ARC-CMKLR1-KD and scrambled virus injected rats was detected (time x treatment, F_(7,98)_ = 2.727, *p* = 0.0125; Figure 3C). This was confirmed when analyzing the area under the curve for energy intake (t_(14)_ = 2.288, *p* = 0.0382; Figure 3D). At 29 days following surgery, no significant differences were found in unfasted glucose levels (Figure 3E).

### Neuropeptides and Inflammatory Markers in the Hypothalamus

**FIGURE 4:**
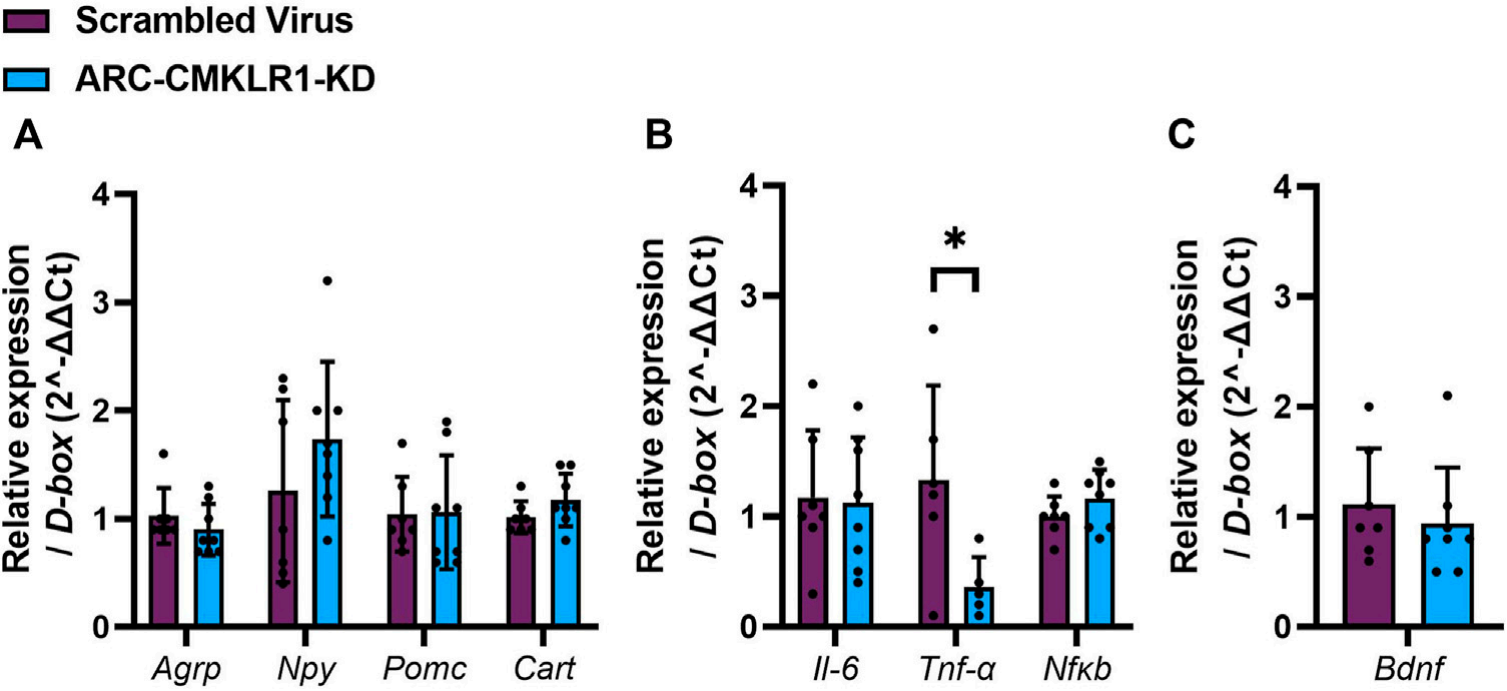
Neuropeptides, inflammatory markers and trophic factor in the hypothalamus. Adult Sprague Dawley rats on high-fat diet received a bilateral injection of a shRNA construct targeted against CMKLR1 or a control construct (*n* = 8/group). After surgery, the rats were maintained on a high-fat diet for 17 days and then switched to chow diet. **(A)** Hypothalamic neuropeptide mRNA expression of Agrp, Npy, Pomc and Cart mRNA remains unaltered in ARC-CMKLR1-KD rats. **(B)** No significant difference was found in Il-6 and Nfκb mRNA levels, but Tnf-α is significantly downregulated in ARC-CMKLR1-KD rats. **(C)** Bdnf mRNA levels did not change. Data are presented as mean ± SD.

Next, we tested if the chemerin-CMKLR1 axis was involved in appetite regulating neuropeptides and inflammatory markers. Initially, hypothalamic neuropeptide gene expression of Agrp, Npy, Pomc and Cart was measured in rats 29 days after knockdown of CMKLR1 in the ARC and showed no difference in gene expression (Figure 4A). Given that chemerin is a potential regulator of inflammation, we then tested gene expression levels of Il-6, Tnf-*α* and Nfκb. While there was no difference of levels Il-6 and Nfκb mRNAs, Tnf-*α* mRNA was significantly reduced in ARC-CMKLR1-KD rats compared to scrambled virus injected rats (t_(9)_ = 2.428, *p* = 0.0381; Figure 4B), suggesting that knockdown of CMKLR1 protects from high-fat induced neuroinflammation. Bdnf, a neurotrophic factor that protects the hypothalamus from high-fat diet induced inflammation, remained unchanged (Figure 4C).

### Gene Expression in White and Brown Adipose Tissue

**FIGURE 5:**
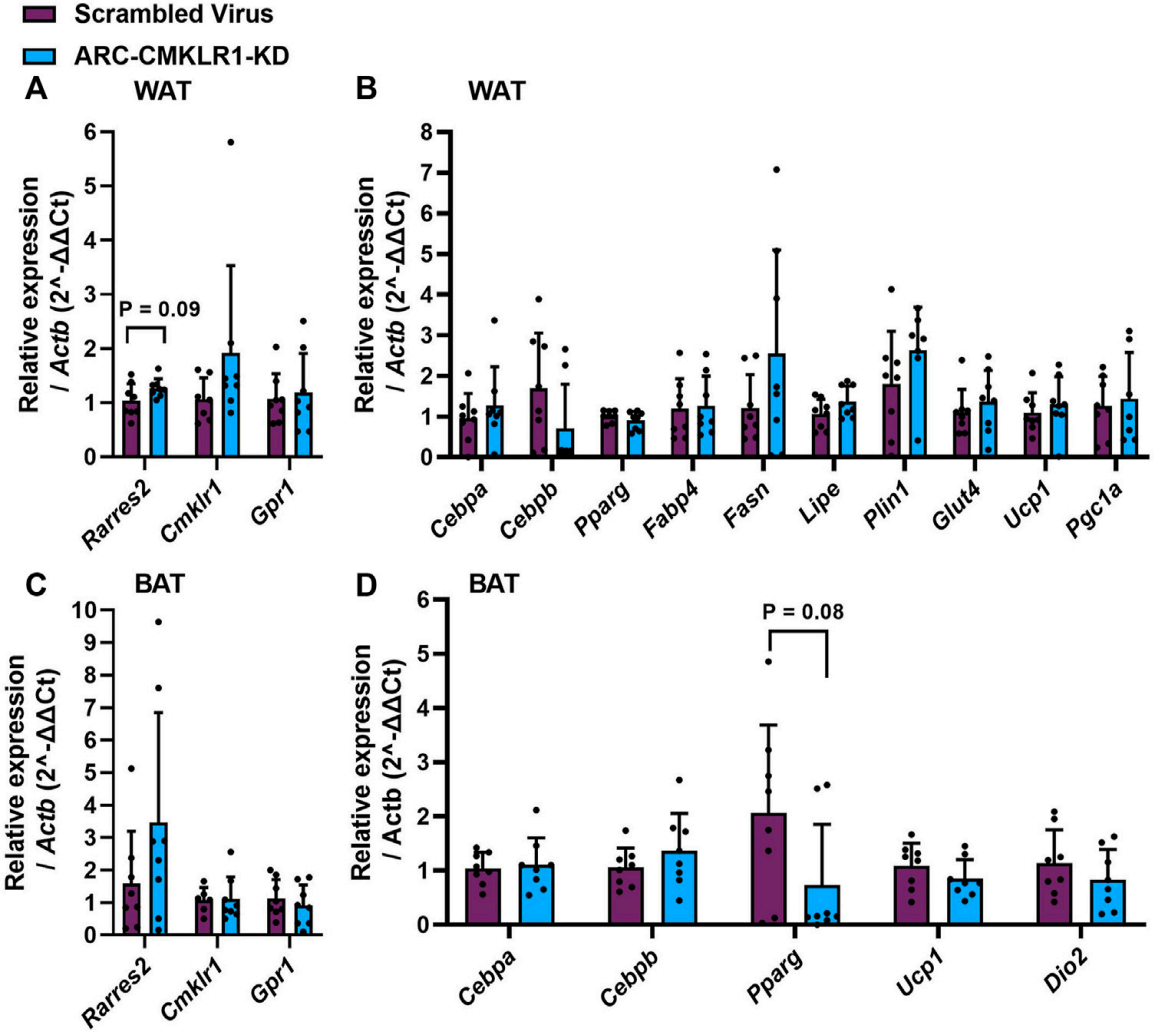
Gene expression of chemerin (Rarres2), its receptors, adipocyte differentiation, function and browning markers in white and brown adipose tissue. Adult Sprague Dawley rats on high-fat diet received a bilateral injection of a shRNA construct targeted against CMKLR1 or a control construct (*n* = 8/group)/After surgery, the rats were maintained on a high-fat diet for 17 days and then switched to chow diet. **(A)** White adipose tissue (WAT) expression of chemerin (Rarres2) and its receptors was unaltered in ARC-CMKLR1-KD rats. **(B)** WAT expression of markers of adipogenesis (Cebpa, Cebpb, Pparg, Fabp4), lipid and glucose metabolism (Fasn, Lipe, Plin1, Glut4), and markers of browning (Ucp1, Pgc1a) remained unaltered in the ARC-CMKLR1-KD rats. **(C)** In brown adipose tissue (BAT) chemerin pathway mRNAs were unaltered in ARC-CMKLR1-KD rats. **(D)** BAT markers of adipogenesis (Cebpa, Cebpb), mitochondrial biogenesis (Pparg) and non-shivering thermogenesis activation (Ucp1, Dio2) were unaltered in ARC-CMKLR1-KD rats.

Chemerin is secreted from and has several known actions in white and brown adipose tissue. Therefore, to determine whether central lentiviral knockdown of CMKLR1 leads to altered chemerin expression, signaling or adipose tissue physiology, qPCR was carried out on key mRNA markers. Chemerin and its receptor (Rarres2, Cmklr1, Gpr1) expression was not significantly altered in white or brown adipose tissue (Figures 5A,C). In white adipose tissue, markers of adipogenesis (Cebpa, Cebpb, Pparg, Fabp4), lipid and glucose metabolism (Fasn, Lipe, Plin1, Glut4), and markers of browning (Ucp1, Pgc1a) remained unaltered in the arcuate nucleus-CMKLR1-knockdown rats (Figure 5B). Similarly, markers of adipogenesis (Cebpa, Cebpb), mitochondrial biogenesis (Pparg) and non-shivering thermogenesis activation (Ucp1, Dio2) were unaltered in arcuate nucleus-CMKLR1-knockdown rats (Figure 5D).

### Short Term Memory Effects of Arcuate Nucleus-CMKLR1-Knockdown

**FIGURE 6:**
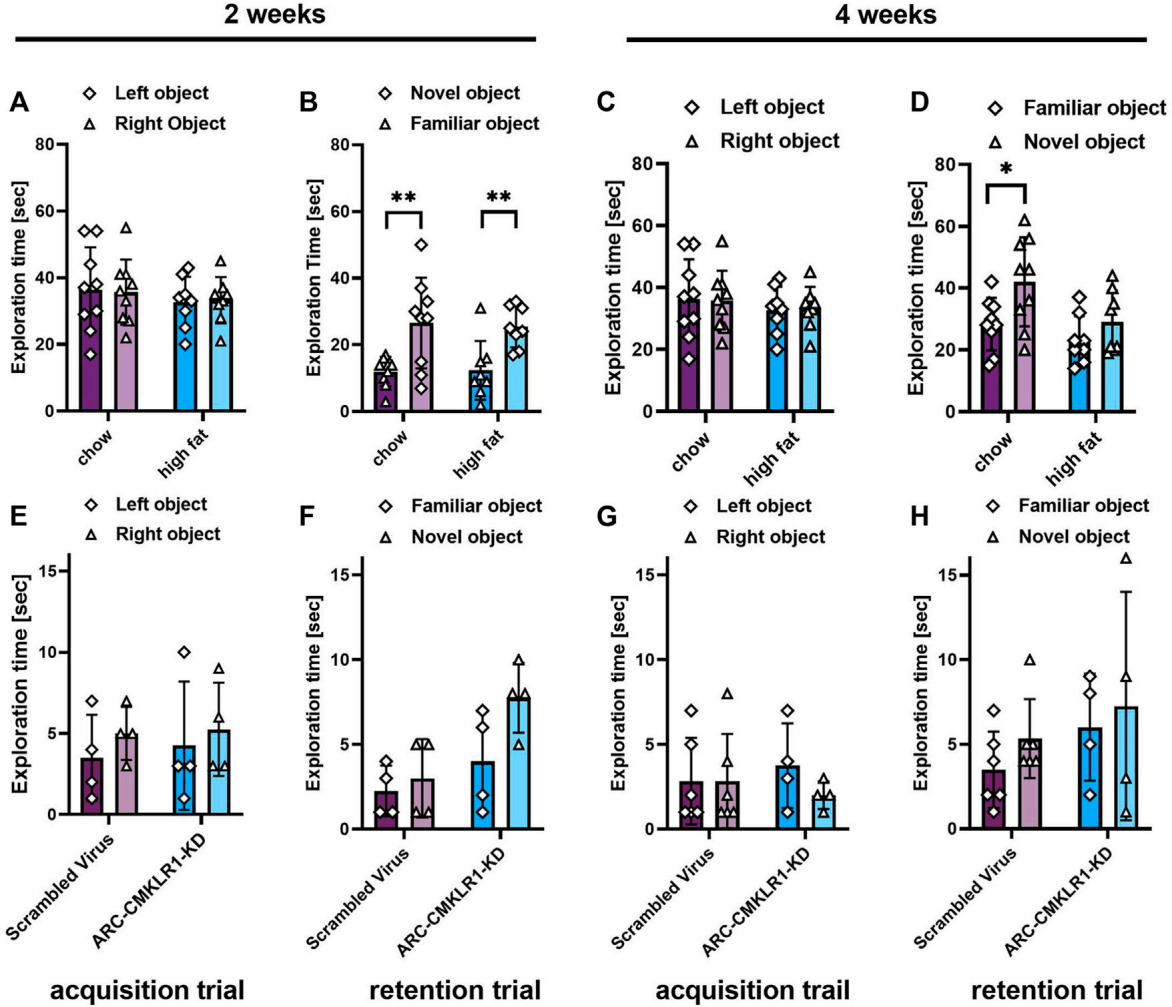
Short term memory effects of CMKLR1 knockdown in the arcuate nucleus. **(A–D)** Initially, performance in the Novel object recognition (NOR) task was tested in Sprague Dawley rats on nutrient-matched chow or high-fat diet. **(E–H)** Sprague Dawley rats on high-fat diet received bilateral injections of a shRNA construct targeted against CMKLR1 or a control construct (*n* = 8/group). After surgery, the rats were maintained on a high-fat diet for 17 days and then switched to chow diet. **(A,E)** Mean exploration time of identical objects in the acquisition trial after 2 weeks. **(B,F)** Mean exploration time of a familiar object and a novel object in the retention trial after 2 weeks. **(C,G)** Mean exploration time of identical objects in the acquisition trial after 4 weeks. **(D,H)** Mean exploration time of a familiar object and a novel object in the retention trial after 4 weeks. Data were analyzed by Student’s paired *t*-test. *p < 0.05, *p* < 0.01. Rats that failed to explore both objects in one or both trials of the tasks were excluded from the analysis, therefore the final group numbers were *n* = 4–9. Data are expressed as mean ± SD.

We then tested whether knockdown of CMKLR1 in the arcuate nuceleus had an impact on Novel object recognition (NOR). Initially, we tested rats on chow and high-fat diet for 2 and 4 weeks (Figure 1C, Experiment 3). One rat was excluded from the analysis as it failed to explore both objects in one trial of the task. After 2 weeks, there was no significant difference spent exploring the two identical objects during the acquisition trial in the chow and high-fat groups (Figure 6A). In the retention trial, both groups explored the novel object significantly more than the familiar object (chow: t_(8)_ = 4.221, *p* = 0.0029; high-fat: t_(7)_ = 03.958, *p* = 0.0055). However, when re-tested after 4 weeks, only the chow diet group explored the novel object significantly more than the familiar object (t*(8)* = 2.445, *p* = 0.0403; Figure 6D). These data show that 4 weeks of high-fat feeding is sufficient to induce an impairment in memory in our rat model. Next, to determine if central knockdown of CMKLR1 protects against high-fat diet inducing short term memory impairment, we conducted NOR test in the arcuate nucleus-CMKLR1-knockdown rats and control rats. Four rats were excluded from the control group and two rats were excluded from the arcuate nucleus-CMKLR1-knockdown group because they failed to explore both objects in one of the trials. As expected, at 2 weeks following surgery for control rats the high-fat diet impaired the ability of rats to differentiate between novel and familiar objects, whereas arcuate nucleus-CMKLR1-knock down rats showed increased exploration of the novel object, however, this was not significant (Figure 6F). 4 weeks following surgery, none of the groups showed a preference (Figure 6H).

## Discussion

Almost all studies of chemerin to date have focused on its release from adipose tissue and its effects on peripheral tissues with the central effects largely overlooked. Here we show for the first time that the chemerin-CMKLR1 axis is functionally important for the central regulation of energy homeostasis and inflammation. Using pharmacological and genetic manipulation approaches, we found a significant inhibition of weight gain after blocking hypothalamic CMKLR1 acutely and chronically.

The small molecule 2-(*α* naphtyl) ethyltrimethylammonium iodide (α-NETA) is a selective CMKLR1 antagonist, which suppresses autoimmune inflammatory response in the central nervous system (Graham et al., 2014). Treatment with α-NETA and α-NETA analogues with structural modifications delay activation of autoimmune encephalomyelitis (Graham et al., 2014; Kumar et al., 2019). Interestingly, α-NETA inhibits fat deposition in the liver and adipose tissue as well as lipid accumulation in the liver of high-fat fed mice and suppresses adipocyte gene expression in WAT demonstrating that α-NETA interferes with lipid metabolism (Xue et al., 2018). To investigate the role of chemerin-CMKLR1 signaling in energy metabolism, we injected α-NETA into the third ventricle of the hypothalamus and measured body weight and food intake. We show that an acute i.c.v. bolus injection of α-NETA reduced body weight in Sprague-Dawley rats after 24 h. We found that both Cmklr1 and Gpr1 mRNA levels were significantly upregulated in α-NETA treated rats, despite previous reports showing that α-NETA selectively inhibits binding of its ligands to CMKLR1 (Graham et al., 2014). The upregulation of Cmklr1 and Gpr1 mRNAs is likely to be in response to decreased activation of these receptors.

To understand the central role of CMKLR1 in energy balance regulation, we then used shRNA mediated knockdown of CMKLR1 in the hypothalamus. It is well established that the neuronal circuitry underlying appetite and body weight regulation is driven by the arcuate nucleus, responsive to external circulating stimuli such as adipokines (Helfer and Stevenson 2020), thus we injected the lentivirus construct bilaterally into the arcuate nucleus. We found a significant inhibition of weight gain in arcuate nucleus-CMKLR1-knockdown rats compared to control rats 28 days after injection. This difference became most apparent after diet change and was accompanied by a reduced energy intake. However, the reduction in energy intake was very subtle, thus it will be interesting to test in future studies whether an increase in energy expenditure causes the inhibition of weight gain. Glucose levels did not change between the two groups. This is consistent with previous reports showing no or little effect on glucose homeostasis in CMKLR1-knockout mice (Rouger et al., 2013; Gruben et al., 2014) whereas GPR1-knockout mice on high-fat diet have increased glucose intolerance compared to wild-type mice (Rourke et al., 2014). Thus, chemerin might control glucose homeostasis exclusively via GPR1. However, in our study glucose levels were measured in unfasted rats thus it is difficult to draw final conclusions. Additionally, there is generally a controversy in the published literature concerning the role of chemerin in regulating insulin sensitivity and glucose uptake (Helfer and Wu 2018). While most studies in humans and animals have shown a positive correlation between chemerin levels and poor glycemic control, no consensus has yet been reached (Léniz et al., 2022).

Previously, we showed that i.c.v. infusion of chemerin into photoperiodic rats altered food intake and body weight, however, chemerin seemed not to directly act through the neuroendocrine appetite regulating pathways (Helfer et al., 2016). Here, we confirm these findings and show that anorexigenic and orexigenic genes in the hypothalamus did not change after blocking CMKLR1 in the arcuate nucleus. However, we found a significant reduction of Tnf-*α* mRNA in the arcuate nucleus-CMKLR1-knockdown rats suggesting that reduced expression of CMKLR1 protects from high-fat diet induced chronic low-grade inflammation. In support, Tnf-*α* and Il-6 mRNA levels are reduced in white adipose tissue in mice lacking the CMKLR1 receptor (Ernst et al., 2012). In our study, we only found a significant effect on Tnf-α, while levels of Il-6 and Nfκ-b mRNAs remained unaltered. Similarly, Bdnf mRNA did not change in the arcuate-nucleus CMKLR1 knockdown rats. No significant effects of arcuate nucleus-CMKLR1-knockdown was detected on mRNA expression of chemerin (Rarres2)*,* markers of adipogenesis, glucose or lipid metabolism, browning (in WAT) or brown adipose function in either WAT or BAT, indicating that effects of the lentiviral knockdown were restricted to the central axis.

In this study, we aimed to knockdown CMKLR1 in rats with high endogenous levels of chemerin. High-fat feeding increases circulating levels of chemerin (Helfer and Wu 2018), but also results in a decline in cognitive performance (Tan and Norhaizan 2019). As a first step, we investigated the time frame necessary to achieve cognitive impairment in our rat model using NOR testing. The NOR test is used as a measure of short-term episodic memory, and we have previously used this test to show acute i.c.v administration of soluble amyloid β oligomers caused robust and enduring deficits (Watremez et al., 2018). In line with the published literature, we found that 4 weeks of high-fat feeding results in cognitive impairment in Sprague Dawley rats (Niepoetter et al., 2021). Hence, we injected the shRNA construct targeting CMKLR1 into rats after 4 weeks of high-fat feeding. As expected, 2 weeks following surgery (i.e. 6 weeks of high-fat feeding) the high-fat diet impaired the ability of control rats to differentiate between novel and familiar objects. At this timepoint, arcuate nucleus-CMKLR1-knockdown rats had a trend for increased exploration of the novel object, suggesting protection from the high-fat diet effects. In support, CMKLR1 deficiency improved memory in a mouse model of Alzheimer’s disease (Zhang et al., 2020). Unfortunately, due to delivery problems, we had to switch the rats to chow diet 17 days after surgery, hence, this makes the lack of difference observed in the 4 weeks NOR test difficult to interpret.

High-fat feeding induced inflammation results in decline in cognitive performance through activation of microglia and secretion of proinflammatory cytokines such as TNF-α (Tan and Norhaizan 2019). The cellular hypothalamic distribution of chemerin and its receptors has not been reported in detail, however, it is known that chemerin and CMKLR1 are expressed in hypothalamic tanycytes (Helfer et al., 2016) and our RNA-seq database indicates expression of chemerin and CMKLR1 in microglia, tanycytes and neurons, with astrocytes so far not tested (Mu et al., 2021). Previously, it was shown that astrocyte and microglia respond to high-fat diet induced hypothalamic inflammation (Baufeld et al., 2016). Our data support the hypothesis that chemerin, elevated by high-fat feeding, activates hypothalamic glial cells causing neuroinflammation. In turn neuroinflammation might cause dysregulation of neuronal activity of neurons in the circuits involved in appetite regulation and body weight. This effect is potentially through hypothalamic tanycytes given that, i.c.v. administration of chemerin causes process extension and proliferation of tanycytes (Helfer et al., 2016). However, it remains to be tested whether chemerin directly activates tanycytes, astrocytes or neurons with subsequent effects on neuroinflammation. Alternatively, hypothalamic inflammation and microglia activation has been shown to contribute to the development of obesity (Valdearcos et al., 2017) hence chemerin might activate microglia to cause neuroinflammation, which causes major morphological changes in tanycytes (Helfer et al., 2016). Either way, it is conceivable that these events disrupt activity of neurons in the arcuate nucleus to regulate appetite, body weight and possibly energy expenditure and glucose homeostasis.

In conclusion, our data demonstrates that CMKLR1 is functionally important for the central effects of chemerin on body weight regulation and implicates the chemerin-CMKLR1 axis in the regulation of whole-body metabolism and cognition. Multiple factors related to modern lifestyle, such as sedentary behavior and overconsumption of calorie-dense food disrupt energy homeostasis and facilitate weight gain. Homeostatic regulation of food intake and energy expenditure results from a complex series of interactions of hormones and neuromodulators in the hypothalamus of the central nervous system to regulate appetite homeostasis. Among the hormones driving this circuitry that link to unwanted effects of modern living is the adipokine chemerin. Since chemerin is upregulated in obesity, our study informs further understanding of fundamental mechanisms underlying lifestyle factors (e.g. high-fat diet) and obesity. This understanding is needed to develop obesity avoidance strategies or appropriate therapies, such as blocking CMKLR1 activity, to address the biggest health challenge of our time.

## Data Availability

The original contributions presented in the study are included in the article/Supplementary Material, further inquiries can be directed to the corresponding author.
