# Supplementary material for: The Chemerin-CMKLR1 Axis is Functionally important for Central Regulation of Energy Homeostasis
Source: Front Physiol. 2022 May 30;13:897105. doi: 10.3389/fphys.2022.897105 (PMC9196942; doi:10.3389/fphys.2022.897105)
Supplement: Supplementary file 1 [file Table1.DOCX]

**Supplementary Table S1: Nucleotide sequences for primer pairs used for gene expression analysis in this study.** WAT: white adipose tissue, BAT: brown adipose tissue**.**

| **Primer** | **Sequence 5’ – 3’** | **Tissue** |
| --- | --- | --- |
| *Actb* forward | CCACACCCGCCACCAGTTCG | WAT, BAT |
| *Actb* reverse | TACAGCCCGGGGAGCATCGT | WAT, BAT |
| *Adipoq* forward | GGAGAGAAGGGAGACGCAGGT | WAT, BAT |
| *Adipoq* reverse | CTTTCCTGCCAGGGGTTC | WAT, BAT |
| *Agrp* forward | CCATATAAGCTCAGGGCACAAG | Hypothalamus |
| *Agrp* reverse | GGTATTGAAGAAGCGGCAGTAG | Hypothalamus |
| *Bdnf* forward | GTCTGTCTGTAAGGGCTAGAATG | Hypothalamus |
| *Bdnf* reverse | GTCTCCTATGAAGCCACCTAATC | Hypothalamus |
| *Cart* forward | CCTCCTTCGGTTCCCATATTTC | Hypothalamus |
| *Cart* reverse | AAGCGAAAGTCCCTCTTCTTC | Hypothalamus |
| *Cebpa* forward | GCCAAGAAGTCGGTGGATAA | WAT, BAT |
| *Cebpa* reverse | CGGTCATTGTCACTGGTCAA | WAT, BAT |
| *Cebpb* forward | CAAGCTGAGCGACGAGTACA | WAT, BAT |
| *Cebpb* reverse | CAGCTGCTCCACCTTCTTCT | WAT, BAT |
| *Cmklr1* forward | GCTCGGACTACATCGTGGACTT | WAT, BAT |
| *Cmklr1* reverse | CGGTGTTCACCGTCTTCTTCATCTTG | WAT, BAT |
| *Cmklr1* forward | TATTCTGCAACAGTGAACAG | Hypothalamus |
| *Cmklr1* reverse | AAGCTGTAGATTACCACCAG | Hypothalamus |
| *D-Box* forward | CGAGGAACAGAAGGATGAGAAG | Hypothalamus |
| *D-Box* reverse | CAGGTGTAAGTCTGAACAAGGA | Hypothalamus |
| *Dio2* forward | TGTCTGGAACAGCTTTCTCC | WAT, BAT |
| *Dio2* reverse | CCATCCGCCGTCTTCTCTG | WAT, BAT |
| *Fabp4* forward | GAAAGAAGTGGGAGTTGGCT | WAT, BAT |
| *Fabp4* reverse | TACTCTCTGACCGGATGACG | WAT, BAT |
| *Fasn* forward | GGATGTCAACAAGCCCAAGT | WAT, BAT |
| *Fasn* reverse | CAGAGGAGAAGGCCACAAAG | WAT, BAT |
| *Glut4* forward | GTAACTTCATCGTTGGCATGG | WAT, BAT |
| *Glut4* reverse | GGCCGAGATCTGGTCAAATG | WAT, BAT |
| *Gpr1* forward | GGAACTCAGCATTCATCACA | WAT, BAT |
| *Gpr1* reverse | GACAGGCTCTTGGTTTCAGC | WAT, BAT |
| *Gpr1* forward | AACTCACTGCTTGTTGTTC | Hypothalamus |
| *Gpr1* reverse | AACAAATAATGCGGTTGTTG | Hypothalamus |
| *Il-6* forward | CCGTTTCTACCTGGAGTTTGT | Hypothalamus |
| *Il-6* reverse | GTTTGCCGAGTAGACCTCATAG | Hypothalamus |
| *Lipe* forward | GCCCTCCAAACAGAAACCC | WAT, BAT |
| *Lipe* reverse | AAATCCATGCTGTGTGAGAA | WAT, BAT |
| *Npy* forward | CCAGACAGAGATATGGCAAGAG | Hypothalamus |
| *Npy* reverse | CAACGACAACAAGGGAAATGG | Hypothalamus |
| *Nfκb* forward | AAAAACGAGCCTAGAGAATTG | Hypothalamus |
| *Nfκb* reverse | ACATCCTCTTCCTTGTCTTC | Hypothalamus |
| *Pgc1a* forward | TGTGCAGCCAAGACTCTGTAT | WAT, BAT |
| *Pgc1a* reverse | TATGTTCGCGGGCTCATTGT | WAT, BAT |
| *Plin1* forward | TTACGGATAACGTGGTAGAC | WAT, BAT |
| *Plin1* reverse | GAGGATTATCGATGTCTTGG | WAT, BAT |
| *Pomc* forward | GAGGTTAAGGAGCAGTGACTAAG | Hypothalamus |
| *Pomc* reverse | GTTCTTGATGATGGCGTTCTTG | Hypothalamus |
| *Pparg* forward | GAAAGACAACAGACAAATCACC | WAT, BAT |
| *Pparg* reverse | GGGGGTGATATGTTTGAACTTG | WAT, BAT |
| *Rarres 2* forward | CGGACATACACGGGACAGAGCTTGA | WAT, BAT |
| *Rarres2* reverse | CAGCTGAGAAGAACAGGTCATCAGCAC | WAT, BAT |
| *Tnf-𝛼* forward | CACACGAGACGCTGAAGTAG | Hypothalamus |
| *Tnf-𝛼* reverse | GAGCAGAGGTTCAGTGATGTAG | Hypothalamus |
| *Ucp1* forward | TGGCCAAGACAGAAGGATTG | WAT, BAT |
| *Ucp1* reverse | GATCTTGCTTCCCAAAGAGG | WAT, BAT |
